# Characterization of a SAM-dependent fluorinase from a latent biosynthetic pathway for fluoroacetate and 4-fluorothreonine formation in *Nocardia brasiliensis*

**DOI:** 10.12688/f1000research.3-61.v1

**Published:** 2014-02-19

**Authors:** Yaya Wang, Zixin Deng, Xudong Qu

**Affiliations:** 1 Key Laboratory of Combinatorial Biosynthesis and Drug Discovery, Ministry of Education, Wuhan University School of Pharmaceutical Sciences, Wuhan, 430071, China

## Abstract

Fluorination has been widely used in chemical synthesis, but is rare in nature. The only known biological fluorination scope is represented by the *fl *pathway from *Streptomyces cattleya* that produces fluoroacetate (FAc) and 4-fluorothreonine (4-FT). Here we report the identification of a novel pathway for FAc and 4-FT biosynthesis from the actinomycetoma-causing pathogen *Nocardia brasiliensis *ATCC 700358. The new pathway shares overall conservation with the *fl* pathway in *S. cattleya*. Biochemical characterization of the conserved domains revealed a novel fluorinase NobA that can biosynthesize 5’-fluoro-5’-deoxyadenosine (5’-FDA) from inorganic fluoride and *S*-adenosyl-l-methionine (SAM). The NobA shows similar halide specificity and characteristics to the fluorination enzyme FlA of the *fl* pathway. Kinetic parameters for fluoride ( *K _m_* 4153 μM, *k _cat_* 0.073 min ^-1^) and SAM ( *K _m_* 416 μM, *k _cat_* 0.139 min ^-1^) have been determined, revealing that NobA is slightly (2.3 fold) slower than FlA. Upon sequence comparison, we finally identified a distinct loop region in the fluorinases that probably accounts for the disparity of fluorination activity.

## Introduction

The introduction of fluorine into organic molecules can often improve their molecular stability and pharmacological properties ^1, 2^. Organo-fluorines have been widely used in pharmaceuticals, diagnostics, agrochemicals, and materials ^3, 4^, and it is estimated that 20–30% of commercial drugs, including many top-sellers, contain fluorine ^5^. The increasing prevalence and success of organo-fluorines have instigated enormous efforts over the past decades in developing methodologies for efficiently introducing fluorine into organic molecules ^5, 6^, however the unique properties of fluorine make these chemical incorporations challenging, usually needing harsh synthetic conditions and giving rise to moderate chemo- or stereo- selectivity ^5, 6^.

**Figure 1.**
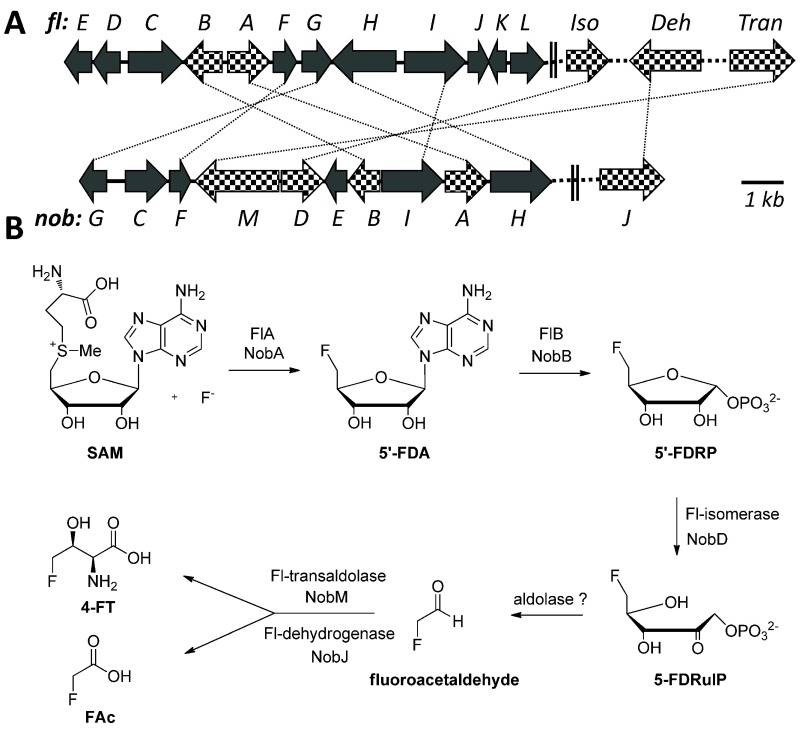
Biosynthetic genes and pathways for FAc and 4-FT biosynthesis. ( **A**) Organization of relevant genes in the *fl* and *nob* pathway, encoding the conversion of fluoride to FAc and 4-FT (crosshatched), and auxiliary function (blackened). Iso: isomerase; Deh: dehydrogenase and Tran: transaldolase. Identical genes are indicated by dotted lines. ( **B**) Proposed biosynthetic pathways to the FAc and 4-FT in the *S. cattleya* and *N. brasiliensis*.

In contrast to chemical synthesis, biocatalytic synthesis or biosynthesis provide better efficacy and selectivity under mild conditions, and can be easily scaled up for industrial production ^7^. Although natural fluorination is rare, efforts in identifying new bio-fluorination machineries are continuing. Pioneered by O’Hagan and co-workers, the natural bio-fluorination pathway to produce fluoroacetate (FAc) and 4-fluorothreonine (4-FT) was biochemically characterized in the actinomycete *Streptomyces cattleya* ^8^. Enzymatic fluorination in *S. cattleya* occurs *via* a fluorine-fixation step that is catalyzed by the unique fluorinase enzyme FlA through the nucleophilic attack of fluoride to SAM ^9– 11^. The resulting fluoro-intermediate 5′-fluoro-5′-deoxyadenosine (5′-FDA) is further converted by five enzymes to give rise to the FAc and 4-FT ( Figure 1A and B) ^8^. This powerful bio-fluorination machinery has been proved very successful in incorporating both ^18^F and ^19^F into nucleotides ^12– 16^ and in producing the anticancer drug candidate fluorosalinosporamide ^17^. Very recently, its enormous potential for producing complex fluorinated molecules was demonstrated by Chang and co-workers ^18^. Using an engaging biosynthetic strategy they were able to achieve site-specific incorporation of FAc into the polyketide backbone of 2-desmethyltriketide lactone. Merging this strategy with the FAC producing pathway, such as the *fl* pathway *in vivo*, could present opportunities to use living cells for the production of acetate-derived fluorinated natural products that include polyketides, fatty acids, terpenoids and steroids.

Synthetic biology approaches need pools of “genetic elements” to mix and match to build and optimize synthetic bio-pathways ^19^. However, the only natural bio-fluorination tool available so far is the *fl* pathway. The limited natural bio-fluorination machinery forms the bottleneck for development of efficient synthetic bio-pathway to produce fluorinated natural products, thus an expansion of the genetic resources of biological fluorination is highly desired. Here, we report the identification of a latent fluorination pathway for FAc and 4-FT biosynthesis from the pathogen *Nocardia brasiliensis* ATCC 700358 and the biochemical characterization of a novel SAM-dependent fluorinase enzyme.

## Materials and methods

### General methods, biochemicals, and chemicals

DNA isolation and manipulation in *Escherichia coli* were performed according to standard methods ^20, 21^. PCR amplifications were carried out on an authorized thermal cycler (Veriti 96 Well, ABI) using PrimeSTAR Max DNA polymerase according to the manufacturer protocol (TaKaRa, Japan). Primer syntheses and DNA sequencing were performed at Genewiz BiotechCo., Ltd. (China). The *E. coli* DH5 *α* cells were purchased from Invitrogen (Carlsbad, CA), and *E. coli* BL21 (DE _3_) cells were purchased from Novagen (Madison). *N. brasiliensis* HUJEG-1 (ATCC 700358) was purchased from the American Type Culture Collection (ATCC), and *S. cattleya* DSM 46488 was provided by Prof. Hong-Yu Ou. Restriction enzymes were purchased from TaKaRa Biotechnology Co., Ltd. (Dalian, China). All other chemicals and reagents were purchased from Santa Cruz Biotechnology, Inc (USA) or Shanghai Sangon Biotech (China) Co., Ltd., unless noted otherwise. Analytical HPLC was carried out on an SHIMADZU LC-20A Prominence HPLC system. LC-MS analysis was conducted on a Thermo Instruments HPLC system connected to a LCQ Fleet electrospray ionization (ESI) mass spectrometer (ThermoFisher Scientific Inc.). NMR data were collected using a Bruker 400 MHz spectrometer.

***Sequence analysis.*** The genetic open reading frame sequences (ORFs) in *N. brasiliensis* were identified using the FramePlot 4.0 beta program ( http://nocardia.nih.go.jp/fp4/). The corresponding proteins were compared with other known proteins in the NCBI databases by using available BLAST methods ( http://www.ncbi.nlm.nih.gov/blast/). Amino acid sequence alignments were performed by using the Strap program ( http://www.bioinformatics.org/strap/).

***Cloning, overexpression and purification of NobA.*** The synthetic gene codon-optimized *nobA* for expression in *E. coli* using the OptimumGene ^TM^ algorithm was sourced from GenScript (Nanjing, China). The optimized sequence exhibited 82% identity to the original sequence and is shown below (codons optimized are underlined, restriction sites are in italics): *GAATTCCATA*TGAC GAC GAC GAATGG CCGCCG CCC GAT TAT TGC GTT TATGAGCGA TCT GGG TATCACCGACGACTC CGTGGCACAGTG TAAGGG TCTGATGCT GAGCGT GTGCCCGGATGT GACGAT TGT TGACATCTG TCA TACCATGCAGCCGTGGGA TGTGGA AGA AGGTGC GCG TTA TAT TGT TGA CCTGCCGCG CCTGTT TCC GGAGGG TACGGT TTTCGC AACCACGAC CTACCCGGC AACCGG TAC GAC CGC ACG TAGCGTCGC TCTGCG TATCGC ACATGCCTC TAA AGGCGG TGC ACG TGG CCAGTGGGC AGG TAGTGGTGC AGGTTTCGAACG CAAGGAAGG CTC ATA TAT TTACATCGC GCCGAACAA TGG TCTGCT GAC GAC CGT TAT TAA AGA ACACGG CTATCTGGAAGCCTACGA AGT TAGCTC TCC GGAAGTCAT CCCGGA ACA ACCGGA ACCGACCTT TTA TTCACGTGA AATGGT GGC ACTGCCGTCGGC TCATCT GGC AGC AGG TTTCCCGCTGGA AAA AGTCGG TCG TCGCCT GGC AGA TGACGA AAT TGT GCG TTT TGAACG CAAGGATCC GGAACTGGT TGCCGA TCACGACCT GGTCGG TTA TGTGACCAACAT TGATCATCCGTT TGGCAACGT TTGGAC GAA TAT CCA CCG TACCGA CCTGGA AAA ACT GGG TGTCGG CTACGG TAC GAA GCTGCG CAT TACCCT GGA TGG TGTGCTGCCGTT TGA ACTGCCGCTG TCCCCGAC CTTCGC AGATGC TGG CGAAATCGG TGC AGC TGT GGC ATATCTG AGTTCCCG TGG TTA CCTGGC ACTGGC ACG TAA TGC TGCG TCGCTGGC GTATCC GTA TAA TCT GAAGGC GGGTAT TTCGGT CCA AGTCAA AGTGGGCT *AAGCTT*. The gene of FlA was PCR amplified from genomic DNA of *S. cattleya* using the forward primer 5′-TTCATATGGCTGCGAACAGCACACGTC-3′ and reverse primer 5′-TTAAGCTTATCAGCGGGCCTCGACCCG-3′. The purified PCR product was ligated to pMD18-T simple (TaKaRa, Japan) following the manufacturer protocol and its fidelity was confirmed by DNA sequencing (multi-color fluorescence-based DNA analysis). The *Nde*I- *Hin*dIII fragments of *nobA* and *flA* were ligated to pET28a to yield the plasmids pWHU2401 and pWHU2402, respectively, which were then been used to overexpress the NobA and FlA proteins as N-terminal 6×His-tagged fusion proteins. The resulting expression plasmids were transformed into *E. coli* BL21 (DE _3_) cells. The cells were grown in 0.8 L of liquid culture (1×Luria broth medium with 50 μg/mL kanamycin) at 37°C to an OD _600_ of 0.6. The cells were allowed to cool room temperature and induced with 0.1 mM isopropyl- *β*-D-thiogalactopyranoside (IPTG) for 10 hours at 25°C. The cells were centrifuged (6000 rpm, 10 minutes, 4°C) resuspended in 15 mL lysis buffer (25 mM HEPES pH 7.5, 300 mM NaCl, 5 mM imidazole, 10% glycerol) and lysed by sonication. Cellular debris was removed by centrifugation (15000 rpm, 30 minutes, 4°C). Two mL Ni-NTA agarose resin was added to the supernatant and the solutions were shaken at 4°C for 1 hour. The protein resin mixtures were loaded into a gravity flow column, and proteins were sequentially eluted with 10 mL Buffer A (25 mM HEPES pH 7.5, 300 mM NaCl, 10% glycerol) supplemented with 50 mM, 100 mM and 300 mM imidazole, respectively. Purified proteins (10 mL) were buffer exchanged by dialysis in 1 L Buffer B (25 mM HEPES, pH 7.5, 50 mM NaCl, 10% glycerol) and concentrated by centrifugation using an Amicon Ultra-4 (10 KDa, GE Healthcare). Proteins purity (90–95%) was evaluated by 12% acrylamide SDS-PAGE. Protein concentrations of the NobA (5 mg mL ^-1^), FlA (4 mg mL ^-1^) and NobA-S158A (25 mg mL ^-1^) were determined by the Bradford method using a BSA calibration curve. The final proteins were flash-frozen in liquid nitrogen and stored at -80°C. Gel filtration chromatography was used to determine the native molecular mass of NobA. Experiments were performed at a flow rate of 1 ml min ^-1^ using an AKTA Purifier FPLC system (Amersham Pharmacia Biotech) and a Superdex 200 GL column (Amersham Pharmacia Biotech). The elution buffer used was 50 mM phosphate buffer (pH 7.8). The native molecular mass of the enzyme was estimated from a calibration curve plotted by using the standard proteins carbonic anhydrase (29 kDa), bovine serum albumin (66 kDa), alcohol dehydrogenase (150 kDa), *β*-amylase (200 kDa), apoferritin (443 kDa), and thyroglobulin (669 kDa).

***In vitro enzymatic assays of NobA.*** Typical assays were performed in 100 μL of 20 mM sodium phosphate buffer (pH 7.5) containing 1 mM SAM, 1–5 μM enzyme and 20 mM NaF or NaCl ^22^. For chlorination reaction, additional 1 μM L-amino acid oxidase (Sigma-Aldrich Co., Ltd.) was added in the reaction mixture. Reactions were incubated at 26°C, quenched by 10 μL 100% trichloroacetic acid (TCA) and the supernatants were subjected to HPLC or LC-HRMS analysis using an analytic Inertsil ODS-3 column (5 μm, 4.6×250 mm, GL Science Inc). HPLC analysis was normally performed with a linear gradient of 5% to 20% CH _3_CN (v/v) over 20 minutes, 20% to 5% CH _3_CN (v/v) over 1 minute, and 5% CH _3_CN (v/v) for further 4 minutes at a flow rate of 1 mL/min under 260 nm. HPLC-ESI-MS analysis was performed as described above. The kinetic parameters for NaF were determined with SAM maintained at a concentration of 0.4 mM and NaF at increasing concentrations from 0 to 20 mM. The kinetic parameters for SAM were determined with a concentration of NaF maintained at 5 mM and SAM at increasing concentrations from 0 to 1 mM. The formation of 5′-FDA was determined by HPLC and quantified using a standard curve of *S*-adenosyl- l-homocysteine (SAH). Each data point represents a minimum of three replicate, end point assays were fitted to the Michaelis-Menten equation by OriginPro 9.0 (OriginLab software, Northampton, MA) to obtain estimates for *k _cat_* and *K _m_*.

***Determination pH and metal ion effect on the activity of NobA and FlA.*** A reaction mixture (100 µl) containing 20 mM NaF, 1 mM SAM in 20 mM sodium acetate (pH 4.5–5.5), sodium phosphate (pH 6.0–7.0), Tris-HCl (pH 7.5–9.0), or Gly–sodium hydroxide (pH 9.5–10.0) buﬀer was prepared. The reactions were initiated by the addition of 5 μM NobA or FlA, incubated at 26°C for 30 minutes where the velocity is in the linear range, and then subjected to HPLC analysis. To measure the metal ion effect on the activity, a group of 100 μl reaction mixtures containing 20 mM NaF, 1 mM SAM in 20 mM Tris-HCl (pH 6.5) buﬀer were spiked with 1 mM diﬀerent divalent metal chloride salts (Mg ^2+^, Mn ^2+^, Fe ^2+^, Cu ^2+^ or Zn ^2+^) or 1 mM ethylenediaminetetraacetic acid (EDTA) to remove the potential associated metal ions of the reaction mixture. Reactions were initiated by addition of 5 μM NobA or FlA, and then incubated at 26°C for 60 min to HPLC analysis. Neither metal ions nor EDTA was added in the negative control reaction mixture. The formation of 5′-FDA was determined by HPLC and quantified using a standard curve of S-adenosyl- l-homocysteine (SAH). Each data point represents a minimum of two replicate, end point assays were fitted to the Excel (Microsoft Corporation) to obtain scatter diagram (PH) and histogram (metal ions) for estimating relative activity.

***Construction and purification of nobA mutant.*** A *nobA* mutant was constructed using a standard PCR method ^21^ and the pWHU2401 as a template. The NobA-S158A mutant was constructed with the primer pair 5′-GACCTTTTATGCACGTGAAATGG-3′ and 5′-CCATTTCACGTGCATAAAAGGTC-3′ according to a standard protocol ^21^. The construct of pWHU2403 was verified by DNA sequencing and was overexpressed in *E. coli* BL21 (DE _3_) as previously described. The expression and purification procedures of recombinant NobA-S158A were carried out in a manner similar to that described for NobA.

## Results and discussion

A putative gene ( *nobA*) encoding a protein with overall 79% identity to the fluorinase FlA was identified from the genome of *N. brasiliensis* ATCC 70035823 ( Figure 1A). By extending the searching region to the up-downstream of *nobA*, we further identified a gene cluster of 10 genes ( *nob*A-I, Figure 1A and Table 1). In this gene cluster, four catalytic genes ( *nobA*, *B*, *D* and *M*) encode the homologues of 5′-FDA synthase, 5′-FDA phosphorylase, 5′-FDRP isomerase and 4-FT transaldolase which are responsible for the conversion of fluoride to 5-FDRibulP and fluoroacetaldehyde to 4-FT in the *fl* pathway) ^8^. Flanked those are genes for encoding auxiliary functions, including regulation ( *nobC, F and G*), transportation ( *nobC*, *H*) and SAM recycling ( *nobE* and *I*), among of which four ( *nobF*, *G*, *I* and *H*) are homologous to the counterparts in the *fl* biosynthetic pathway. The overall identity to the *fl* pathway genes suggested that the *nob*A cluster might be related to the 4-FT and FAc biosynthesis.

**Table 1.** Putative orfs in the *nob* biosynthetic pathway from *N. brasiliensis* (accession number KF963271).

Gene	Size	Protein homologue and origin	Identity/similarity (%)	Proposed function
G	223	*flG* ( CAJ20008), from *S. cattleya* DSM 46488	39/60	DNA binding protein (regulatory)
C	340	SCAT_p0565 ( YP_004919857), from *S. cattleya* DSM 46488	69/79	Permease
F	191	*flF* ( YP_004913663), from *S. cattleya* DSM 46488	59/73	DNA binding protein (regulatory)
M	659	*4-FTase* ( YP_006051324), from *S. cattleya* DSM 46488	62/74	4-fluorothreonine transaldolase
D	334	*5-FDRP isomerase* ( YP_006053901), from *S. cattleya* DSM 46488	45/56	5-FDRP isomerase
E	191	adenine phosphoribosyltransferase ( WP_016574607), from *S. albulus*	57/66	adenine phosphoribosyl transferase
B	292	*flB* ( CAJ20005), from *S. cattleya* DSM 46488	57/70	5'-fluoro-5'-deoxy- adenosine phosphorylase
I	502	*flI* ( YP_004913660), from *S. cattleya* DSM 46488	74/85	*S*-adenosyl- l- homocysteine hydrolase
A	300	*flA* ( YP_004913664), from *S. cattleya* DSM 46488	81/89	5'-fluoro-5'- deoxyadenosine synthase
H	476	*flH* ( YP_004913661), from *S. cattleya* DSM 46488	47/64	Na ^+^/H ^+^ antiporter
J	507	*Aldehyde dehydrogenase* ( YP_004910482.1), from *S. cattleya* DSM 46488	78/87	Aldehyde dehydrogenase

Besides the four catalytic genes described above, other two genes encoding an aldolase and a dehydrogenase to mediate the conversion of 5-FDRibulP to fluoroacetaldehyde and fluoroacetaldehyde to FAc are necessary. A homology search for the fluoroacetaldehyde dehydrogenase in the *N. brasiliensis* resulted in the identification of a gene with 78% identity ( *nobJ*, YP_006807765.1; Figure 1A and Table 1) from a remote site in the genome, suggesting that *N. brasiliensis* has the potential to produce FAc. Unlike the others, the gene encoding aldolases in the *fl* pathway is still elusive. Recently, four aldolase genes were identified from *S. cattleya* by genome sequencing and two of them were thought to encode the conversion of 5-FDRibulP to fluoroacetaldehyde ^24^. However, in the *N. brasiliensis* genome we could not find identical homologues by BLAST searching. Because this reaction can also be catalised by fuculose aldolase ^8^ enzyme, we searched for fuculose aldolase homologues, with no success. Instead, five putative aldolase genes ( YP_006810507.1, YP_006812798.1, YP_006805383.1, YP_006812725.1 and YP_006809408.1) were identified in *N. brasiliensis* based on the gene annotation in IMG database ( https://img.jgi.doe.gov/cgi-bin/w/main.cgi). We then compared the genetic sequences of the putative aldolases to their homologues in *S. cattleya*. Interestingly, two of these aldolase genes ( YP_004910624 and YP_004919742.1) which were previously not idenfied in *S. cattleya* shared sequence homology to the aldolase genes ( YP_006810507.1 and YP_006812798.1 respectively) of *N. brasiliensis*. Their homology (both have 62% identity) is in line with the average identity (58%) between the counterpart genes of *nob* and *fl* pathway, suggesting that these genes are probably involved in the FAc biosynthetic pathway.

Previous studies revealed the thioesterase FlK confers self-immunity to the FAc in the *fl* pathway ^25, 26^. Inactivation of the *flK* gene does not affect cell growth in 2 M NaF ^27^, however can prevent production of fluorometabolites ^27^. Interestingly, we were not able to find the *flK* homologue in *N. brasiliensis*, although there are a few genes encoding thioesterases. To test the ability of *N. brasiliensis* to produce fluorometabolites, the bacteria were subjected to fermentation with a supplement of 2 mM NaF as a fluorine source ^28^. Despite the *N. brasiliensis* grows normally, no new fluorine signal in the culture except fluoride could be detected by F-NMR (data not shown). The lack of fluorometabolites might be caused by improper fermentation conditions or deficiency of the FlK homologue in the genome. *In vivo* gene manipulations including promoter activation and heterologous complementation of the *flK* in *N. brasiliensis* were also attempted, but failed due to the inaccessibility of genetic system.

**Figure 2.**
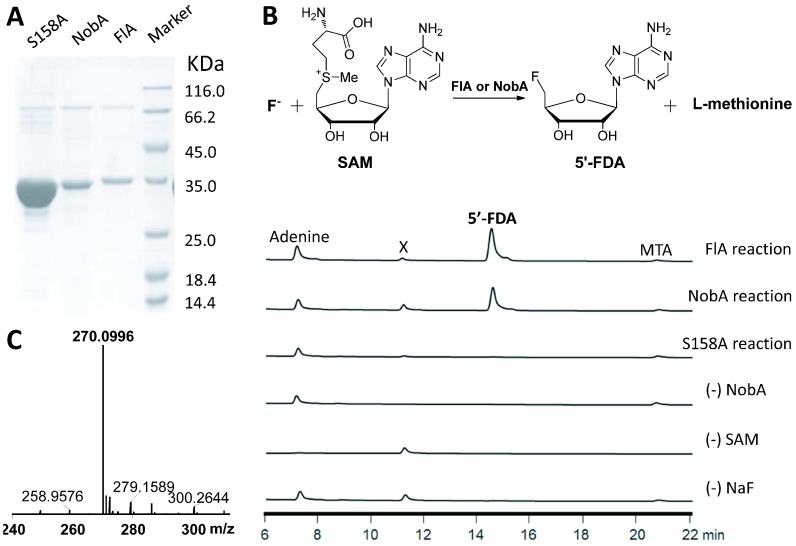
Fluorinase catalyzed conversion of fluoride and SAM to 5′-FDA and l-methionine. **A**) SDS-PAGE analysis of purified recombinant proteins. The expected sizes of NobA-S158A (34.7 kDa), NobA (34.7 kDa) and FlA (34.6 kDa) are indicated. Lane M, protein molecular weight standards. **B**) HPLC showing NobA-catalyzed production of 5′-FDA with various control reactions. Adenine and MTA ( *S*-methyl-5′-thioadenosine) are from the degradation of SAM, and X is an impurity associated with NobA. **C**) HRMS confirming the identity of 5′-FDA. The observed value (m/z [M+H] ^+^ 270.1002) is consistent with the calculated value (m/z [M+H] ^+^ 270.0996).

**Figure 3.**
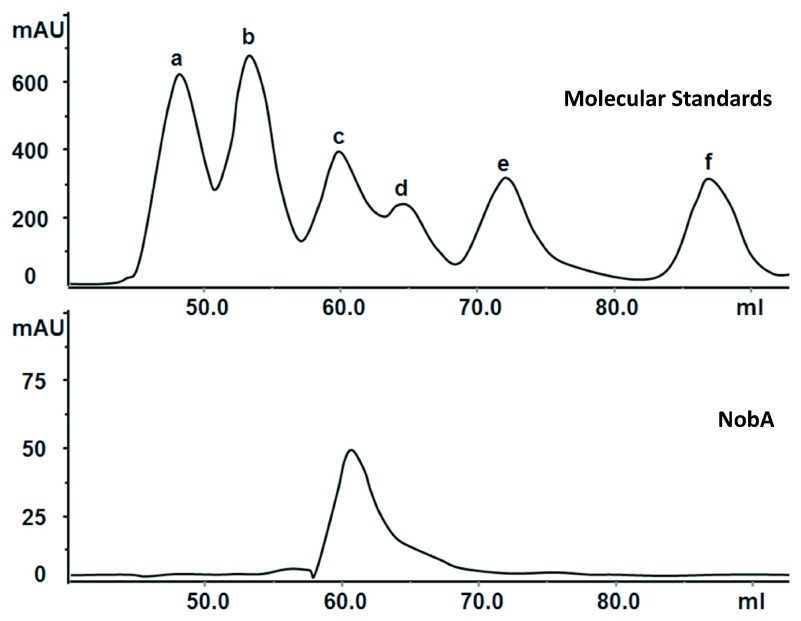
Determination of the native molecular mass of NobA by gel filtration chromatography. Standard proteins (a–f) used are the following: thyroglobulin (669 kDa), apoferritin (443 kDa), *β*-amylase (200 kDa), alcohol dehydrogenase (150 kDa), bovine serum albumin (66 kDa) and carbonic anhydrase (29 kDa). NobA has similar molecular weight to *β*-amylase (200 kDa) suggesting it naturally presence as a hexamer.

Since the *in vivo* studies were inaccessible, we turned to *in vitro* characterization. The 5′-FDA synthase which catalyzes the unique fluorine-fixation reaction is one of the most interesting enzymes for the communities of enzymology and synthetic biology, thus we focused on the biochemical characterization of the NobA enzyme. The *nobA* gene was first codon optimized, cloned as an *N*-terminal 6×His tagged protein, and then expressed and purified from *E. coli* ( Figure 2A). In our experiment, the expression yield of NobA (7.9 mg/L) was lower than the yield of FlA (9.2 mg/L), and like FlA it natively occurs as a hexamer ( Figure 3) ^10^. The fluorination activity of the recombinant protein was assessed in parallel with FlA which was utilized to produce the 5′-FDA as a control. After the reaction, the assay mixtures were quenched by 10% trichloroacetic acid (TCA) and subjected to HPLC analysis. A product strictly dependent on the addition of SAM, NaF and NobA was detected, and its identity was finally confirmed to be 5′-FDA by both HRMS and comparison to the product of FlA ( Figure 2B and C). These results, taken together, indicate that NobA, like FlA, can confer fluorine fixation, and that the *nob* pathway might be related to FAc and 4-FT biosynthesis.

**Figure 4.**
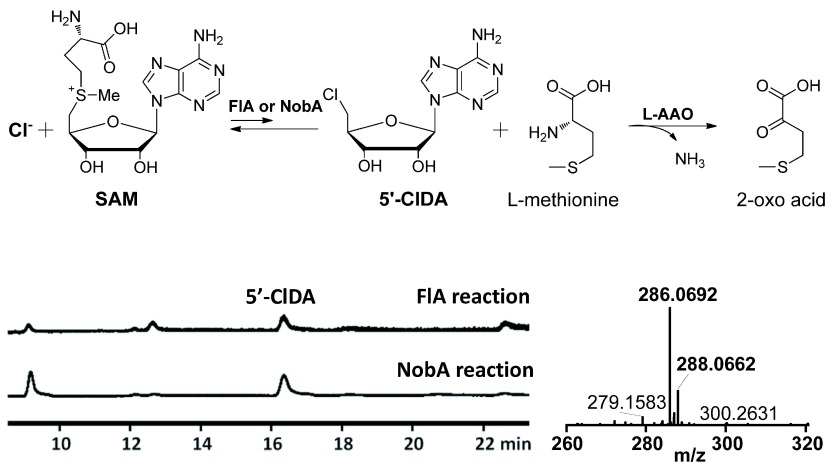
Fluorinase catalyzed conversion of chloride and SAM to 5′-ClDA and l-methionine in the presence of L-amino acid oxidase. The production of 5′-ClDA was determined by HPLC and HRMS. The observed values (m/z [M+H] ^+^^35^Cl = 286.0692, ^37^Cl = 288.0662) are consistent with the calculated values (m/z [M+H] ^+^^35^Cl = 286.0707, ^37^Cl = 288.0677).

**Figure 5.**
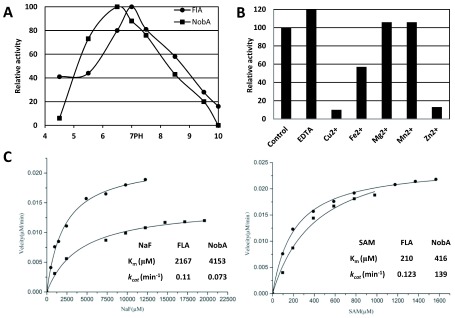
pH and metal ions effect, and kinetic analysis of the fluorination reaction catalyzed by NobA (■) and FlA (●). ( **A**) pH effect on NobA and FlA activity. The optimum activity of NobA at pH 6.5 and FlA at pH 7.0 were individually set as 100%. ( **B**) Metal ion effect on NobA activity. The activity of negative control was set as 100%. ( **C**) Kinetic analysis of the fluorination reaction. When the concentration of NaF was varied, the SAM was fixed at 0.4 mM, otherwise when the SAM was varied, the NaF was fixed at 5 mM.

With a new fluorinase in hand, we next compared the enzymatic properties of the protein. It has been previously reported that in the presence of L-amino acid oxidase (L-AAO), FlA can utilize Cl ^-^ but not Br ^-^ or I ^-^ to form the 5′-Chloro-5′-deoxyadenosine (5′-ClDA) ^22^. Similarly, we observed that NobA was also able to perform this reaction with a similar reactivity ( Figure 4). The optimal pH of NobA was determined as 6.5, slightly lower than FlA at 7.0 ( Figure 5A). NobA shows no obvious effects on fluorination reactivity in the presence of metal ions, instead 1 mM Cu ^2+^ or Zn ^2+^ can severely inhibit its activity ( Figure 5B). The activity can be slightly increased by adding EDTA, suggesting the fluorination reaction is metal independent. The optimal temperature for fluorination reactivity is 37°C, however at this temperature SAM degradation is also expedited. In contrast, as 26°C can provide acceptable activity and slow down the degradation of SAM, this temperature was chosen throughout our kinetic studies. Although previous FlA kinetic studies have been reported ^10, 29^, the data are inconsistent. Thereby, to precisely compare their reactivities, both NobA and FlA were kinetically measured in this study ( Figure 5C). The *K _m_* values of NobA were 4153 μM for NaF and 416 μM for SAM, about twice the values of FlA (2167 μM for NaF and 210 μM for SAM). The turnover numbers ( *k _cat_*) of NobA were 0.073 min ^-1^ for NaF and 0.139 min ^-1^ for SAM, which were similar to the values of FlA (0.11 min ^-1^ for NaF and 0.123 min ^-1^ for SAM). The average *k _cat_*/ *K _m_* of FlA (5.07×10 ^-5^ μM ^-1^ min ^-1^ for NaF and 5.86×10 ^-4^ μM ^-1^ min ^-1^) outnumbers 2.3 fold to the values of NobA (1.75×10 ^-5^ μM ^-1^ min ^-1^ for NaF and 3.3×10 ^-4^ μM ^-1^ min ^-1^ for SAM), and suggests that NobA is less efficient than FlA.

**Figure S1.**
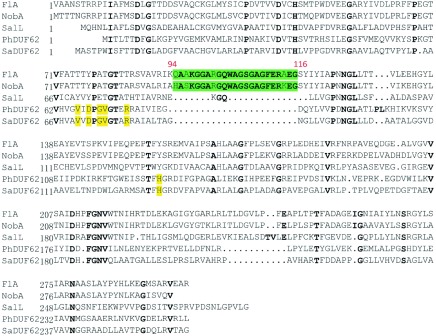
SAM-dependent haloginases and hydrolases of the DUF62 family. The residues highlighted in yellow are critical for SAM hydrolysis. The unique loops of fluorinase are highlighted in bright green. Accession numbers for DUF62 proteins are SalL from *Salinispora tropica* ( ABP73643), PhDUF62 from *Pyrococcus horikoshii* OT3 ( NP_142440) and SaDUF62 from *S. arenicola* CNS-205 ( ZP_01648926).

**Figure S2.**
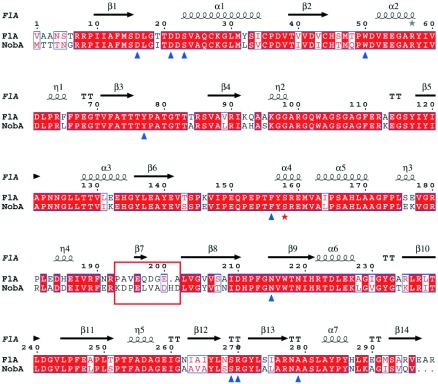
Sequence comparison between FlA and NobA. The secondary structure of FlA (PDB No. 2V7V) is shown on the top. Critical residues (D16, D21, S23, W50, Y77, F156, N215, S269, R270 and A279) for SAM and fluoride binding are labeled by blue triangle). Residue S158 for fluoride desolvation is labeled by red pentagram. The distinct loop region (R192-L202, FlA numbering) which is bracketed by rectangle.

Similar to FlA, NobA retains the conservation of the unique 23-residue loop, which is related to site architecture and fluoride selectivity, and lacks the critical residues for SAM hydrolysis ( Figure S1) ^30, 31^. This unique feature confirms that fluorinases form a distinct group of enzymes which differ from other SAM-binding members in the DUF62 enzyme family. The overall identity of the fluorinases is very high (79% identity), and only a region between the R192 to L202 (FlA residue numbering) is distinct ( Figure S2). Interestingly, in the crystal structure of FlA, this region constitutes a loop which links the N- and C-terminal domains to form an interface where the SAM and fluoride are bound ^11^. The variety of the loop region can probably influence the domain interaction and binding affinities of the substrates, and this might be one of the reasons for the retardation observed for NobA. Moreover, the residue S158 of FlA plays critical role in fluoride binding and desolvation ^10, 32^. Interestingly, this residue also exists in the NobA ( Figure S2). To confirm the essentiality of S158 for NobA activity, this residue was further mutated into an alanine ( Figure 2A). The resulting recombinant protein NobA-S158A completely lost fluorination activity ( Figure 2B) and demonstrates it is essential for NobA activity. This observation also indicates that NobA shares an identical catalytic mechanism employed by NobA.

Actinomycetoma is a zoonotically chronic, granulomatous and subcutaneous tissue infection caused by actinomycetes that may often lead to amputation or death ^33^. *N. brasiliensis* is the major causative pathogen of the actinomycetoma infections in Mexico ^23, 33^. Its pathogenic mechanism currently is unclear. FAc is a highly toxic poison (LD _50_ = 0.1 mg/kg mouse ≈ 1.2 μM) ^34^. In the human body it is first converted into the anabolite (-)-erythro-fluorocitrate (EFc), which exerts its toxicity by irreversibly inhibiting aconitase to cause cell death (IC _50_ = 0.01 μM) ^35, 36^. Interestingly, our research revealed the *N. brasiliensis* has the genetic potential to produce FAc from fluoride. In human blood, the fluoride concentration is normally around 0.13 μM and can increase to 3.2 μM in some fluorine-polluted areas ^37^, which indicates *N. brasiliensis* can potentially produce FAc at levels that cause severe cytotoxicity and lead to development of the actinomycetoma.

**Figure S3.**
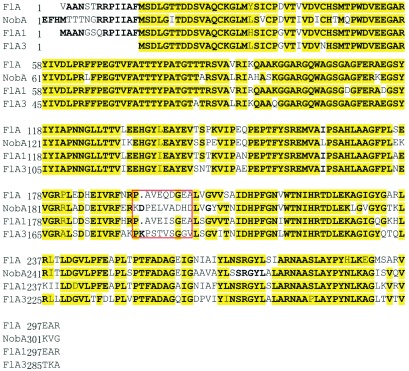
Sequence comparison between FlA, NobA, FlA1 and FlA3. Conserved residues are indicated by bright yellow. The distinct loop region (R192-L202, FlA numbering) is highlighted by rectangle. FlA and NobA are from this study; FlA1 is from *Streptomyces sp.* MA-37 and FlA3 is from *Actinoplanes sp.* N902-109 ^39^.

In summary, we identified a latent pathway for FAc and 4-FT biosynthesis from the actinomycetoma pathogen *N. brasiliensis* ATCC 700358. Comparative analysis of the genomes of *N. brasiliensis* and *S. cattleya* revealed two common aldolase genes that were putatively involved in the FAc and 4-FT biosynthesis. By *in vitro* characterization, we confirmed a novel fluorinase, NobA, that can biosynthesize 5′-FDA from inorganic fluoride and SAM. This new fluorinase has similar substrate selectivity and characteristics to the homologue FlA, with a slightly less efficiency of reaction (2.3 fold). The lower reactivity might be attributed to the presence of a distinct loop region in the sequences of FlA and NobA, based on comparative sequence analysis. During the revision of this manuscript, O’Hagan and co-workers published the identification of the same fluorinase and two other fluorinases from *S. sp* MA37, *Actinoplanes sp* N902-109 ^38^. These fluorinases are highly conserved (76–79% identity to each other) but all have the distinct loop region which is identified by this study ( Figure S3). This feature indicates that fluorinases are a unique protein family whose evolution is also unusual, majorly concentrated at the interface of N- and C- domains. Moreover, by searching the homologue of the putative *S. cattleya* 5-FDRibulP aldolases ( YP_004910624 and YP_004919742.1, identified in this study) in the geome of *A. sp* N902-109 results identification of an aldolase (YP_007950157.1). It shares 65% identity to the aldolase from *S. cattleya* ( YP_004910624) and *N. brasiliensis* ( YP_006810507.1) indicating that this common homologue is likely responsible for the conversion of 5-FDRibulP. The discovery of new fluorinases and biosynthetic pathways increases the genetic resource of bio-fluorination and will benefit the future development of synthetic bio-pathways to produce fluorinated natural products.

## Data availability

Data sets showing the fluorinase catalysed conversions of fluoride and chloride to 5′-FDA and 5′-ClDA are publicly available in ZENODO.

ZENODO: Conversion of fluoride and chloride catalysed by SAM-dependent fluorinase in *Nocardia brasiliensis*, doi: 10.5281/zenodo.8339 ^38^.
