# Supplementary material for: Characterization of a SAM-dependent fluorinase from a latent biosynthetic pathway for fluoroacetate and 4-fluorothreonine formation in Nocardia brasiliensis
Source: F1000Res. 2014 Feb 19;3:61. [Version 1] doi: 10.12688/f1000research.3-61.v1 (PMC3999930; doi:10.12688/f1000research.3-61.v1)
Supplement: Peer review history

# Peer review: Referee response for version 1

**Published:** 2014-03-24

**Authors:** Cormac Murphy

**Affiliations:** 1 Center for Synthesis and Chemical Biology, University College Dublin, Dublin, Ireland

The paper confirms the very recent work of Deng *et al.* (2014) reporting a fluorometabolite biosynthetic gene cluster in the genome of Nocardia brasiliensis, the expression of a codon-optimised fluorinase gene (nobA) in E. coli, and the in vitro assay of fluorination/chlorination activity with SAM and fluoride/chloride.  Overall the paper is well written, the experimental approach is appropriate, with the exception of the kinetic assays as described by Changsheng Zhang, and the conclusions justified. The kinetics of both the enzymes investigated differ somewhat from those reported by Deng *et al., *and the authors should account for these differences, particularly in the K _m_ values for SAM.

I have read this submission. I believe that I have an appropriate level of expertise to confirm that it is of an acceptable scientific standard.

# Peer review: Referee response for version 1

**Published:** 2014-03-05

**Authors:** Changsheng Zhang

**Affiliations:** 1 South China Sea Institute of Oceanology, Chinese Academy of Sciences, Guangzhou, China

This is an interesting article dealing with the characterization of a fluorinase from Norcardia brasiliensis. This research was obviously carried out independently and met publishing scientific standard, so it deserves publication in F1000Research, although similar work has been published ( Deng *et al*., 2014). This work mainly focused on the comparative studies on the fluorination of SAM to form 5’-FDA by two fluorinases NobA and FlA. Most of the experiments were well executed, except for the kinetic determination for these two enzymes. I suggest the authors reinvestigate the kinetic parameters, since it was not appropriate to determine the Km for NaF with a “maintaining” concentration of SAM at only 0.4 mM (the author stated that Kms for SAM of FlA and NobA were 0.2 mM and 0.4 mM, respectively). A saturation concentration is normally around 5 X Km.

I have read this submission. I believe that I have an appropriate level of expertise to confirm that it is of an acceptable scientific standard.
